# Effects of Xylooligosaccharides on Lipid Metabolism, Inflammation, and Gut Microbiota in C57BL/6J Mice Fed a High-Fat Diet

**DOI:** 10.3389/fphar.2021.791614

**Published:** 2021-11-22

**Authors:** Fang Li, Qian Li, Yu Zhang, Xianrong Zhou, Ruokun Yi, Xin Zhao

**Affiliations:** ^1^ Chongqing Collaborative Innovation Center for Functional Food, Chongqing Engineering Research Center of Functional Food, Chongqing Engineering Laboratory for Research and Development of Functional Food, Chongqing University of Education, Chongqing, China; ^2^ Department of Nuclear Medicine, Chongqing University Central Hospital/Chongqing Emergency Medical Center, Chongqing, China; ^3^ College of Food Science, Southwest University, Chongqing, China

**Keywords:** AMPK pathway, gut bacteria, gut-liver axis, lipid metabolism, xylooligosaccharides

## Abstract

Xylooligosaccharide (XOS) is a source of prebiotics with multiple biological activities. The present study aimed to investigate the effects of XOS on mice fed a high-fat diet. Mice were fed either a normal diet or a high-fat diet supplemented without or with XOS (250 and 500 mg/kg), respectively, for 12 weeks. The results showed that the XOS inhibited mouse weight gain, decreased the epididymal adipose index, and improved the blood lipid levels, including triglyceride (TG), total cholesterol (TC), and low-density lipoprotein cholesterol (LDL-C) levels. Moreover, XOS reduced the activity of alanine aminotransferase (ALT) and aspartate aminotransferase (AST), and alleviated the damage to the liver caused by the high-fat diet. XOS also reduced hyperlipidemia-associated inflammatory responses. Additionally, quantitative real-time polymerase chain reaction results showed that XOS intervention activated the AMP-activated protein kinase (AMPK) pathway to regulate the fat synthesis, decomposition, and *β* oxidation; upregulated the mRNA expression levels of carnitine palmitoyl transferase 1 (CPT-1), peroxisome proliferator–activated receptors α (PPAR-α), and cholesterol 7-alpha hydroxylase (CYP7A1); and downregulated the mRNA expression levels of acetyl-CoA carboxylase (ACC), CCAAT/enhancer-binding protein alpha (C/EBPα), and lipoprotein lipase (LPL). On the other hand, XOS enhanced the mRNA expression levels of zonula occludens-1 (ZO-1), occludin, and claudin-1 in the small intestine; increased the strength of the intestinal barrier; and optimized the composition of the intestinal microbiota. Therefore, it was concluded that XOS regulated the intestinal barrier, changed the intestinal microecology, and played an important role in preventing hyperlipidemia through the unique anatomical advantages of the gut–liver axis.

**GRAPHICAL ABSTRACT:**
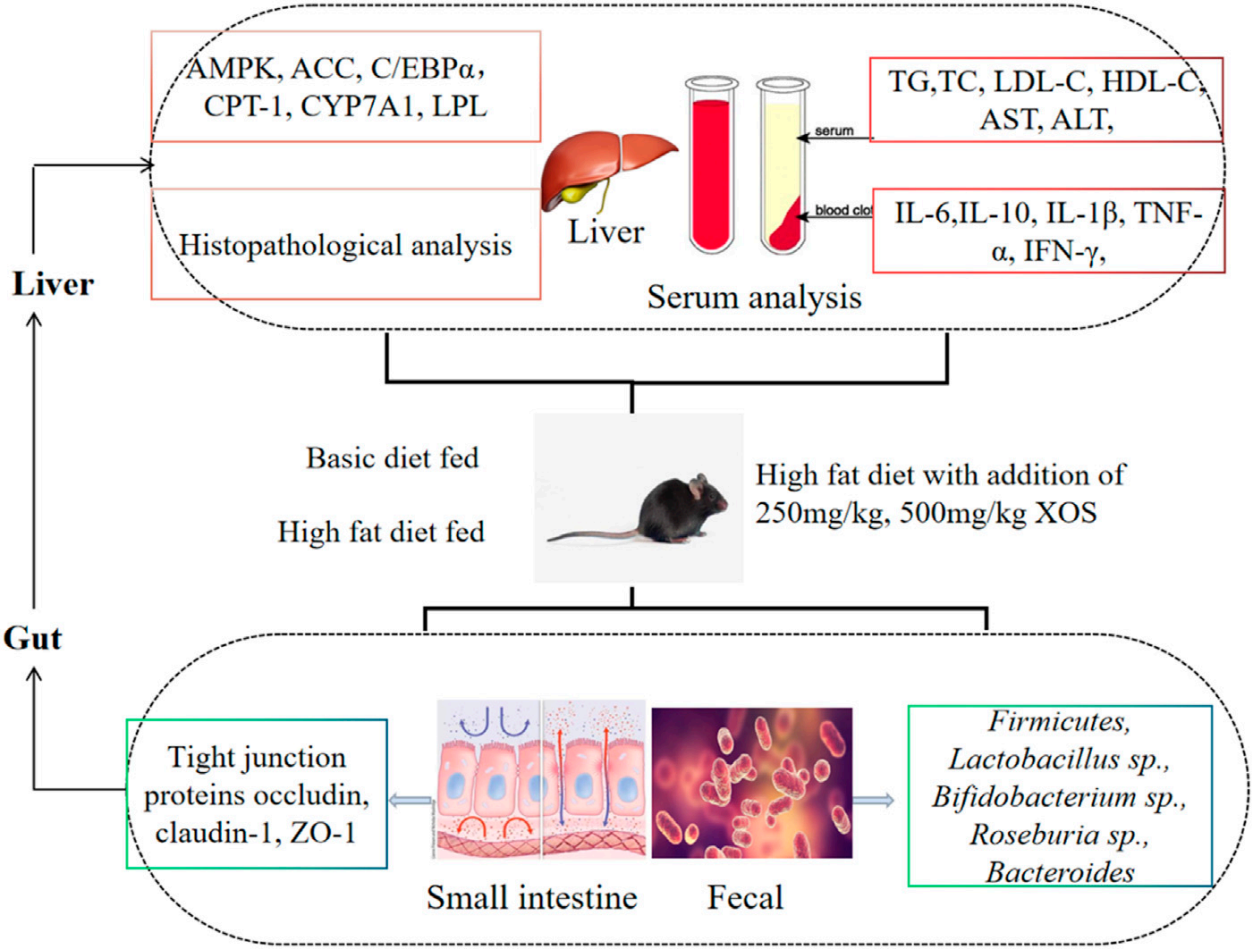


## Introduction

Hyperlipidemia is a metabolic disease worldwide and also the main cause of cardiovascular- and cerebrovascular-related diseases. The latest data show that about 30 million people die from related diseases caused by hyperlipidemia every year in the world, which are also important causes of fatty liver, high blood sugar levels, and hypertension (Oishi et al., 2018). Common hyperlipidemia usually manifests as elevated serum total cholesterol (TC) levels, elevated triglyceride (TG) levels, or decreased high-density lipoprotein cholesterol (HDL-C) levels (Samuels et al., 2002). Many reasons account for hyperlipidemia. Mainly diet, lifestyle, sex, age, genetics, and other factors have led to varying degrees of hyperlipidemia. A healthy lifestyle, including moderate exercise, good work and rest habits, and a healthy diet, can effectively control blood lipid levels; however, it is often difficult for people to adhere to this lifestyle (Kaur, 2014). Therefore, the use of drugs to treat hyperlipidemia is often the most direct intervention for patients.

Xylooligosaccharide (XOS) exists in the cell walls of many grains as nondigestible carbohydrates, improve intestinal microecology, and promote lipid, protein, and mineral metabolism compared with statins. XOS is a polymer composed of 2–7 xylose molecules connected by β-1,4 glycosidic bonds, which prevent XOS from being decomposed by endogenous digestive enzymes (Geraylou et al., 2012; Guerreiro et al., 2015). However, XOS can be used by beneficial microorganisms in the animal intestine, especially the large intestine, and selectively promote the proliferation of microorganisms such as lactic acid bacteria in the host’s intestine. At the same time, they inhibit the growth of harmful bacteria (Guerreiro et al., 2015). This change in the intestinal microbiota is related to the changes in short-chain fatty acids (SCFA) in the gastrointestinal tract, intestinal inflammation, the permeability of the intestinal barrier, and health (Geraylou et al., 2012; Spiljar et al., 2017). A study found that XOS supplements reduced the accumulation of visceral fat and regulated the composition of the cecal microbiota in mice (Spiljar et al., 2017). Another study found that XOS increased the abundance of *Bifidobacteria* in mice fed a high-fat diet and increased the content of SCFA, especially butyric acid (Berger et al., 2021).

The absorption of dietary nutrients increases, fatty tissue accumulates in the liver, and fatty acid metabolism decreases, resulting in decreased secretion of intestinal mucosal barrier factors, such as GLP-1 and GLP-2, and impaired intestinal mucosal integrity (Lore`ne et al., 2017). The intestinal barrier is the body’s first line of defense against harmful substances entering the human circulation from the intestine. The integrity of the intestinal mucosal barrier is essential for maintaining intestinal function and the stability of the internal environment (Yu and Yang, 2009). The anatomical location and function of the liver and intestine are closely related. In addition, researchers put forward the gut–liver axis theory (Christine et al., 2018), which believed that both acute and chronic metabolic diseases were heavily dependent on the function of intestinal flora. Accumulating evidence showed that the imbalance of intestinal flora was another important endogenous factor in the process of abnormal blood lipid metabolism caused by the continuous intake of nutrients (Paola et al., 2019; Zhao et al., 2019). The animal model of a high-fat diet showed that obesity causes intestinal flora imbalance, damaging intestinal integrity and further inducing low-grade inflammation (Chen et al., 2018). A high-fat diet increases the permeability of the intestinal tract, thus increasing certain intestinal endotoxin lipopolysaccharide (LPS) into the blood circulation and leading to metabolic inflammation of the body. Therefore, for abnormal blood lipid metabolism caused by a high-fat diet, one of the most effective strategies is to improve the intestinal microbiota. Dietary intervention with added functional food ingredients promotes lipid metabolism and increases the systemic anti-inflammatory activity of the intestinal tract.

Based on domestic and foreign literature, although many studies have reported on the lipid-lowering and inflammation-reducing abilities of XOS, few studies have systematically analyzed the role of XOSs between intestinal flora and lipid metabolism/inflammation from the direction of the gut–liver axis. In this study, we hypothesized that gavage XOS (250 and 500 mg/kg) mainly acts on the gut–liver axis by regulating the intestinal mucosal barrier function and improving the composition of intestinal microbes, thereby regulating the expression of genes related to liver fatty acid metabolism, reducing inflammation of the body, and achieving regulatory effect on the x dyslipidemia. To verify this hypothesis, C57BL/6J mice were given two different concentrations of XOS by gavage for 12 weeks. Then, intestinal barrier function, intestinal microbes, lipid metabolism–related gene and protein in the liver, histopathology, and inflammation were analyzed to determine the potential mechanism and provide a new method for preventing hyperlipidemia.

## Materials and Methods

### Source of Materials/Animals

Forty 6-week-old male C57BL/6J mice (average weight 21.2 ± 0.2 g) were used in this study and were purchased from the Animal Experiment Center of Chongqing Medical University (Chongqing, China). Basic diet (12.79% fat, 66.67% carbohydrate, and 20.54% protein) and high-fat diet (60.65% fat, 21.22% carbohydrate, and 18.14% protein) were provided by the National Research Center, Jiangsu, China. Xylooligosaccharides (product number: ml015750) were purchased from Shanghai Enzyme Biotechnology Co., Ltd. (Shanghai, China). The purity was 98%, of which xylobiose accounts for 40%.

### Treatment of Animals

The mice were divided into normal group, vehicle group, XOS-H group, and XOS-L group, with 10 mice in each group. They were raised in an environment with a temperature of 25 ± 2°C under a 12 h light–dark cycle. After 1 week of acclimatization, the body weight was recorded. The mice in the normal group were given a basic diet as a control experiment, while the mice in the remaining groups were given a high-fat diet to establish a hyperlipidemia model. The normal group was fed a basic diet, and the remaining groups were fed a 60% fat calories. XOS was dissolved in water [250 mg/kg (b•w); 500 mg/kg (b•w)] and was given to the XOS-L and XOS-H groups, and the intragastric dose was 10 ml/kg. The normal and vehicle groups were given the same amount of normal saline. The experiment lasted for 12 weeks. During this period, the mice were allowed ad libitum access to food and drink. The litter was changed every 3 days, the water was changed every 2 days, and the diet was changed every other day to avoid fast oxidation of the high-fat diet. The mice were weighed every week (12 weeks). After gavage intervention, all mice fasted and drank freely for 16–24 h. The blood, liver, epididymal fat, small intestine tissue, and cecal contents were collected and stored at −80°C. The weight of epididymal fat tissue was determined using the following equation (Li F. et al., 2019):

Epididymal fat index = Epididymal fat mass (g)/Mouse body mass (g) ×100

All the experiments were performed according to the Directive 2010/63/EU and People’s Republic of China National Standard (GB/T 35892-2018), laboratory animal guidelines for ethical review of laboratory animal welfare, and institutional rules considering animal experiments. Meanwhile, the study protocol was approved by the Ethics Committee of Chongqing Collaborative Innovation Center for Functional Food (202009123B, Chongqing, China).

### Biochemical Analysis

The collected blood samples were centrifuged at 3,000 rpm for 10 min, and then the serum in the upper part was taken. The TC, TG, low-density lipoprotein cholesterol (LDL-C), HDL-C, alanine aminotransferase (ALT), and aspartate aminotransferase (AST) levels were analyzed using the kit (Nanjing Institute of Bioengineering, Nanjing, China).

### Enzyme-Linked Immunosorbent Assay

The collected blood samples were centrifuged at 3,000 rpm for 10 min, and then the serum in the upper part was taken. The enzyme-linked immunoassay kit (Shanghai Enzyme Biotechnology Co., Ltd., Shanghai, China) was used to analyze the levels of interleukin-6 (IL-6), interleukin-10 (IL-10), interleukin-1β (IL-1β), interferon-gamma (IFN-γ), and tumor necrosis factor-alpha (TNF-α).

### Histological Analysis

The liver and epididymal fat (about 1 cm^2^) were immersed in tissue fixative for 24 h. Then, they were cleaned, waxed, sectioned, and finally stained with hematoxylin and eosin. The morphology and structure were observed under a biological microscope (BX43; Olympus, Tokyo, Japan) (Li W. et al., 2019). The adipocyte diameter was analyzed using the ImageJ software (U.S. National Institutes of Health, Bethesda, MD, United States). The relative adipocyte size was compared with that of the normal group.

### Real-Time Quantitative Polymerase Chain Reaction

The liver and epididymal fat of mice in the normal, vehicle, and high- and low-dose xylooligosaccharide groups were selected as the test objects of key lipid metabolism–related genes, namely adenosine 5′-monophosphate (AMP)-activated protein kinase (AMPK), acetyl-CoA carboxylase (ACC), carnitine palmitoyl transferase 1 (CPT-1), CCAAT/enhancer-binding protein alpha (C/EBPα), cholesterol 7-alpha hydroxylase (CYP7A1), lipoprotein lipase (LPL), peroxisome proliferators-activated receptors alpha (PPAR-α), and peroxisome proliferator–activated receptor gamma (PPAR-γ). Real-time reverse transcriptase–quantitative polymerase chain reaction (RT-qPCR) was used to analyze the effect of XOS on lipid metabolism pathways in mice at the transcription level. The small intestine tissue was used to determine the relative expression of tight junction (TJ) proteins, namely occludin, claudin-1, and zonula occluden-1 (ZO-1). The primer sequences are shown in Table 1.

**TABLE 1:** Sequences of primers.

Gene name	Sequence
AMPK	Forward: 5′-GTC​AAA​GCC​GAC​CCA​ATG​ATA-3′
	Reverse: 5′-CGT​ACA​CGC​AAA​TAA​TAG​GGG​TT-3′
ACC	Forward: 5′-AGT​GAT​GGT​GGC​CTG​CTC​TTG-3′
	Reverse: 5′-AGC​AGA​CGG​TGA​GCG​CAT​TA -3′
C/EBPα	Forward: 5′-GCG​GGA​ACG​CAA​CAA​CAT​C-3′
	Reverse: 5′-GTC​ACT​GGT​CAA​CTC​CAG​CAC-3′
PPAR-γ	Forward: 5′-AGG​CCG​AGA​AGG​AGA​AGC​TGT​TG-3′
	Reverse: 5′-TGG​CCA​CCT​CTT​TGC​TGT​GCT​C-3′
CPT-1	Forward:5′- TGG​CAT​CAT​CAC​TGG​TGT​GTT-3′
	Reverse: 5′-GTC​TAG​GGT​CCG​ATT​GAT​CTT​TG-3′
PPAR-α	Forward: 5′-AAC​ATC​GAG​TGT​CGA​ATA​TGT​GG-3′
	Reverse: 5′-CCG​AAT​AGT​TCG​CCG​AAA​GAA-3′
LPL	Forward: 5′-TTG​CCC​TAA​GGA​CCC​CTG​AA-3′
	Reverse: 5′-TTG​AAG​TGG​CAG​TTA​GAC​ACA​G-3′
CYP7A1	Forward: 5′-GCT​GTG​GTA​GTG​AGC​TGT​TG-3′
	Reverse: 5′-GTT​GTC​CAA​AGG​AGG​TTC​ACC-3′
Claudin-1	Forward: 5′-GGG​GAC​AAC​ATC​GTG​ACC​G-3′
	Reverse: 5′-AGG​AGT​CGA​AGA​CTT​TGC​ACT-3′
Occludin	Forward: 5′-TGA​AAG​TCC​ACC​TCC​TTA​CAG​A-3′
	Reverse: 5′-CCG​GAT​AAA​AAG​AGT​ACG​CTG​G-3′
ZO-1	Forward: 5′-GCC​GCT​AAG​AGC​ACA​GCA​A-3′
	Reverse: 5′-TCC​CCA​CTC​TGA​AAA​TGA​GGA-3′
β-actin	Forward: 5′-CAT​GTA​CGT​TGC​TAT​CCA​GGC-3′
	Reverse: 5′-CTC​CTT​AAT​GTC​ACG​CAC​GAT-3′
*Total Bacteria*	Forward:5′- ACT​CCT​ACG​GGA​GGC​AGC​AGT-3′
	Reverse: 5′-ATT​ACC​GCG​GCT​GCT​GGC-3′
*Firmicutes*	Forward: 5′-GCG​TGA​GTG​AAG​AAG​T-3′
	Reverse: 5′-CTA​CGC​TCC​CTT​TAC​AC-3′
*Bacteroidetes*	Forward: 5′-ACG​CTA​GCT​ACA​GGC​TTA​ACA-3′
	Reverse: 5′-ACG​CTA​CTT​GGC​TGG​TTC​A-3′
*Lactobacillus* sp.	Forward:5′- CAC​CGC​TAC​ACA​TGG​AG-3′
	Reverse: 5′-AGC​AGT​AGG​GAA​TCT​TCC​A-3′
*Bifidobacterium* sp.	Forward:5′- TCGCGTCYGGTGTGAAAG-3′
	Reverse: CCACATCCAGCRTCCAC-3′
*Roseburia* sp.	Forward: 5′-GCGGTRCGGCAAGTCTGA-3′
	Reverse: 5′-CCTCCGACACTCTAGTMCGA-3′

The TRIzol method was used to extract RNA from the liver, epididymal fat, and small intestine tissue by the method proposed by Mu et al. (2020). The RNA concentration was determined, and a nucleic acid analyzer was used to determine the purity value between 1.8 and 2.0 before proceeding with subsequent tests. Then, a reverse transcription kit (product number: K1622) was used to reverse-transcribe the high-quality RNA into cDNA, which was stored at −80°C. Finally, the cDNA was used as a template and placed with fluorescent dyes in a fluorescent quantitative PCR machine. β-actin gene was used as a reference gene, and the 2^−ΔΔCt^ relative quantification method was used to calculate the expression of each group of genes (Tu et al., 2019).

### Western Blot Analysis

The liver tissue (100 mg) was weighed, and 1 ml of protein lysate was added for protein extraction using the kit (BC3710). The protein concentration was quantified using the bicinchoninic acid (BCA) protein concentration determination kit (PC0020). Electrophoresis was performed after protein denaturation. Then, the proteins were electrotransferred onto PVDF membrane. The transfer conditions were as follows: 22 V and 45 min. A 5% skimmed milk powder sealing solution was prepared with 1× Tris-buffered saline containing 0.1% Tween-20 solution (TBST), and the membrane was immersed into it completely and incubated at room temperature on a shaker for 1 h. The membrane was incubated with primary antibodies overnight and washed five times with 1× TBST solution, followed by incubation with the secondary antibody for 1 h at room temperature. Also, the PVDF membrane was cleaned five times again. Then, an iBright FL1000 imaging system was used for developing the images. Finally, the ImageJ software (U.S. National Institutes of Health, Bethesda, MD, United States) was used to quantify protein expression.

### Relative Abundance of Selected Gut Bacteria in Cecal Content

The fecal genomic DNA extraction kit (Tiangen Biotech Co., Ltd., Beijing, China) was used to extract bacteria from the cecal contents of mice. The specific operation steps were completed following the kit (DP328) instructions. The extracted cecal DNA was used as a template and the total bacteria as an internal reference. An RT-qPCR amplification was performed to determine the relative abundance of *Lactobacillus* sp., *Bifidobacterium* sp., *Roseburia* sp., *Firmicutes*, and *Bacteroides* in the intestines of each group of mice. The primer sequences are shown in Table 1.

### Statistical Analysis

Data were expressed as mean ± SD. The data were processed using Graph Pad Prism 7.0 software (Graph Pad Software, La Jolla, CA), and the comparisons among the groups were made by the analysis of ANOVA. *p < 0.05, **p < 0.01, ***p < 0.001 compared with the Vehicle group.

## Results

### Body Weight and Epididymal Fat Change in Animal Experiments

**FIGURE 1:**
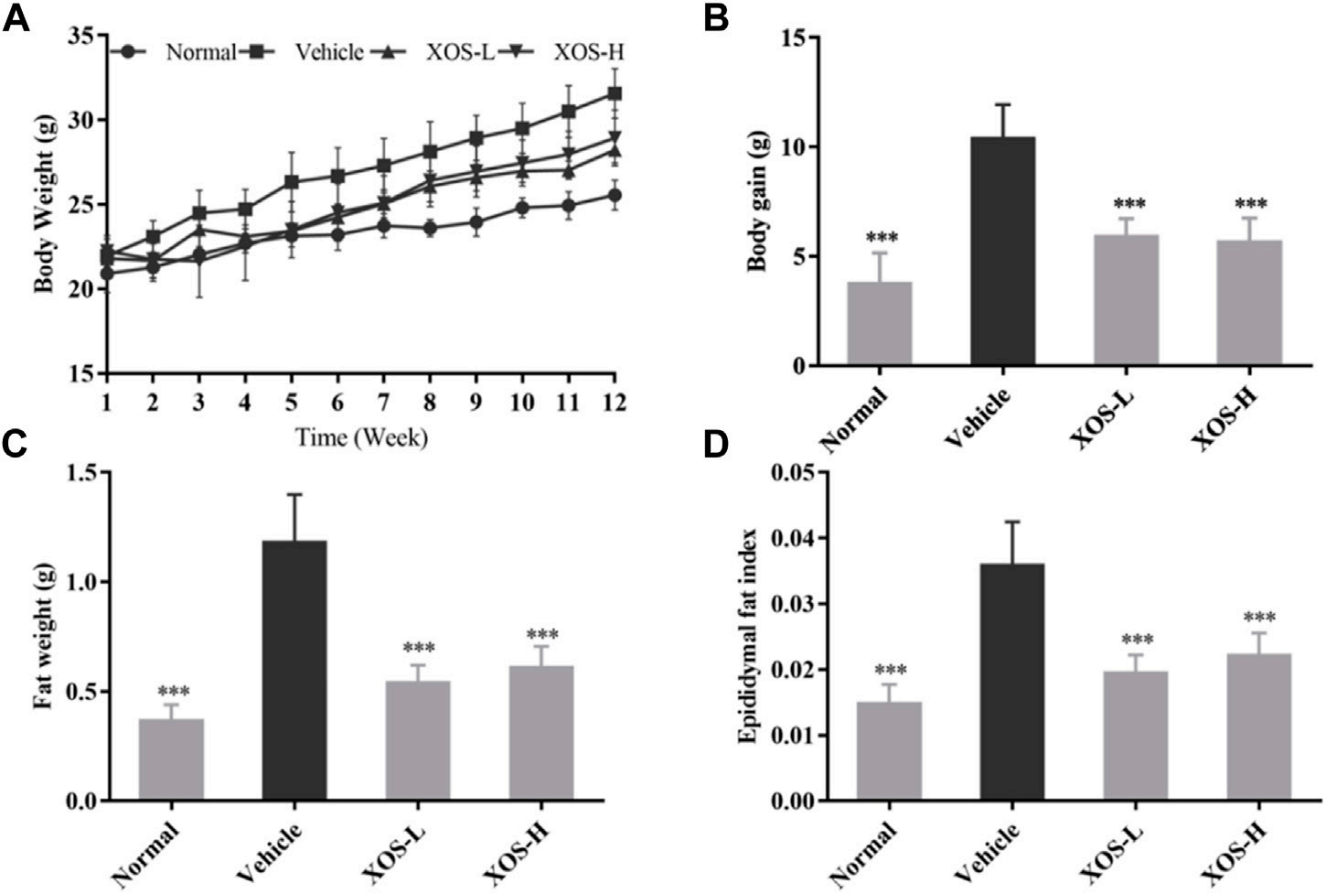
Effect of XOS supplementation on **(A)** Body weight curve; **(B)** weight gain; **(C)** Epididymal fat weight; **(D)** Epididymal fat index. XOS-L: mice treat with XOS 250 mg/kg, XOS-H: mice treat with XOS 500 mg/kg. ***p < 0.001 compared with the Vehicle group.

During the 12-week period, the weight of the normal mice fed a basic diet increased from 21 to 25 g on average, with a weight gain rate of 19%. The weight of the vehicle mice fed a high-fat diet increased from 22 to 32 g, with a weight gain rate of 45% (Figures 1A,B). Compared with the vehicle group, after 12 weeks of XOS intervention, the body weight of the mice in the XOS-H group significantly reduced by 16.32%. Moreover, the vehicle group had the largest epididymal fat index and maximum fat accumulation (Figures 1C,D). After 12 weeks of intervention with high and low doses of XOS, the epididymal fat of the mice was reduced by 54.2 and 48.3%, respectively, showing that XOS could reduce fat accumulation caused by the high-fat diet.

### Serum Lipid and Lipoprotein Levels

The changes in blood lipid and lipoprotein levels in mice were observed after 12 weeks of feeding (Table 2). Compared with the normal group, the contents of TG, TC, and LDL-L in the vehicle group increased significantly by 67.8, 47.4, and 129.4%, respectively, indicating that the high-fat diet successfully induced dyslipidemia in mice. XOS supplementation attenuated blood lipid and lipoprotein levels. The TG, TC, and LDL-C levels decreased by 45.7, 21.9, and 41.0%, respectively, in the XOS-H group. However, XOS did not significantly increase HDL-C levels in mice fed a high-fat diet.

**TABLE 2:** Lipid level of each group at the end of the experiment.

Group	Normal	Vehicle	XOS-L	XOS-H
TC (mmol/L)	2.53 ± 0.24^***^	3.73 ± 0.33	2.77 ± 0.17^***^	2.91 ± 0.24^***^
TG (mmol/L)	0.56 ± 0.08^***^	0.94 ± 0.0.6	0.45 ± 0.09^***^	0.51 ± 0.11^***^
HDL-C (mmol/L)	0.36 ± 0.06^***^	0.14 ± 0.03	0.16 ± 0.06	0.17 ± 0.06
LDL-C (mmol/L)	0.34 ± 0.11^***^	0.78 ± 0.07	0.52 ± 0.11^***^	0.46 ± 0.07^***^

Data are means ± SD. Values in the same column with different letter superscripts mean significant difference (*p* < 0.05). XOS-L: mice treat with XOS 250 mg/kg, XOS-H: mice treat with XOS 500 mg/kg. ***p < 0.001 compared with the Vehicle group.

### Serum AST and ALT Activities

**FIGURE 2:**
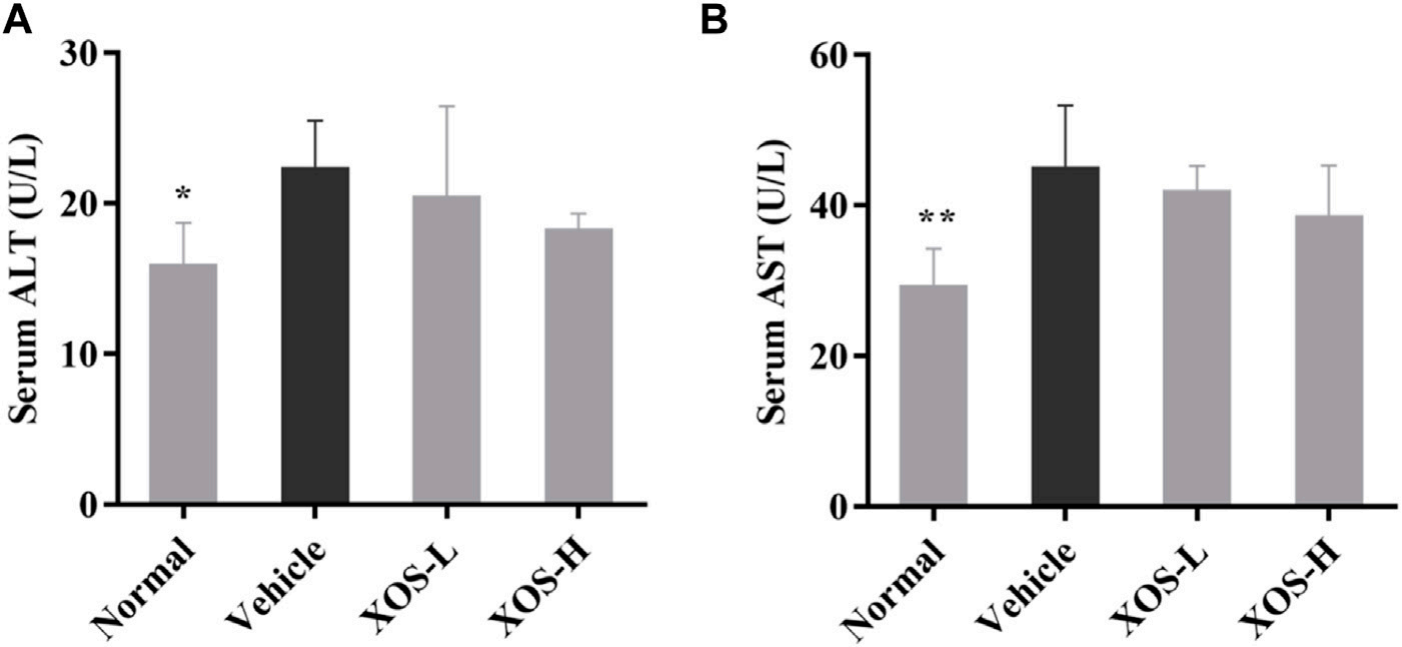
Effect of XOS supplementation on **(A)** serum ALT level; **(B)** serum AST level. XOS-L: mice treat with XOS 250 mg/kg, XOS-H: mice treat with XOS 500 mg/kg. *p < 0.05, **p < 0.01 compared with the Vehicle group.

ALT and AST, as important amino acid transaminase in the body, are markers of liver cell damage. As shown in Figure 2, the ALT and AST activities increased significantly in the serum of the vehicle group of mice induced by the high-fat diet compared with the normal group of mice (*p* < 0.05), indicating that the high-fat diet caused a certain degree of damage to the liver of mice. XOS intervention effectively inhibited the increase in ALT and AST activities caused by a high-fat diet and relieved liver damage.

### Serum Cytokine Levels

**FIGURE 3:**
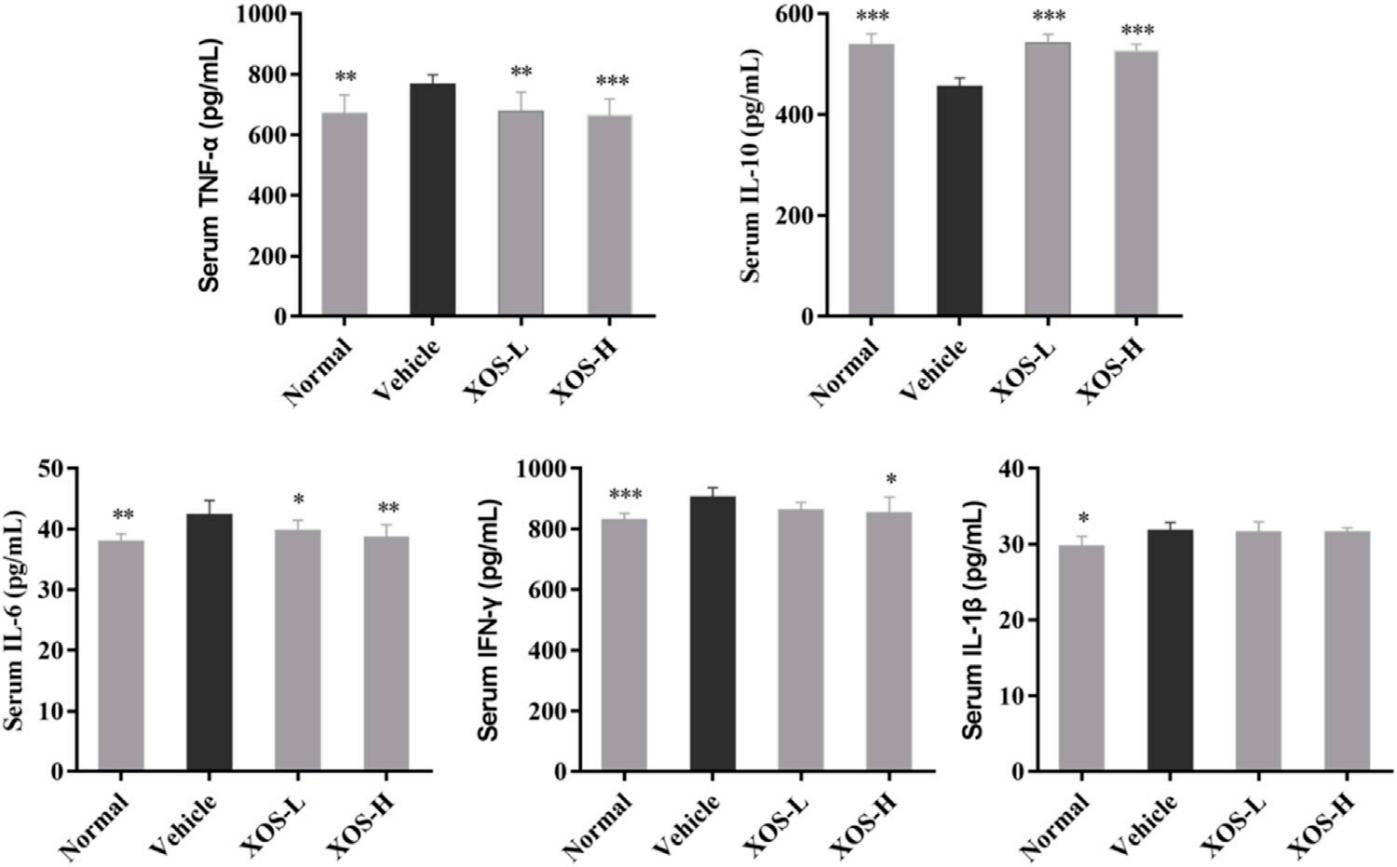
Effect of XOS supplementation on serum Cytokines level in mice. XOS-L: mice treat with XOS 250 mg/kg, XOS-H: mice treat with XOS 500 mg/kg. *p < 0.05, **p < 0.01, ***p < 0.001 compared with the Vehicle group.

Serum levels of IL-6, IFN-γ, TNF-α, and IL-1β were the highest and the IL-10 levels were the lowest in the vehicle group after 12 weeks of administration, while the normal group mice showed the opposite trend, as shown in Figure 3. XOS decreased the effects of inflammation stimulation on mice, including the levels of serum inflammatory factors TNF-α, IFN-γ, and IL-6 decreased, the IL-10 levels increased. However, no significant difference was found between the interventions with different doses of XOS. This showed that XOS increased the level of anti-inflammatory factor IL-10 and reduced the levels of pro-inflammatory factors TNF-α, IFN-γ, and IL-6 in the serum, thus exerting a regulatory effect on the blood lipid levels of mice with hyperlipidemia.

### Histopathological Analysis

**FIGURE 4:**
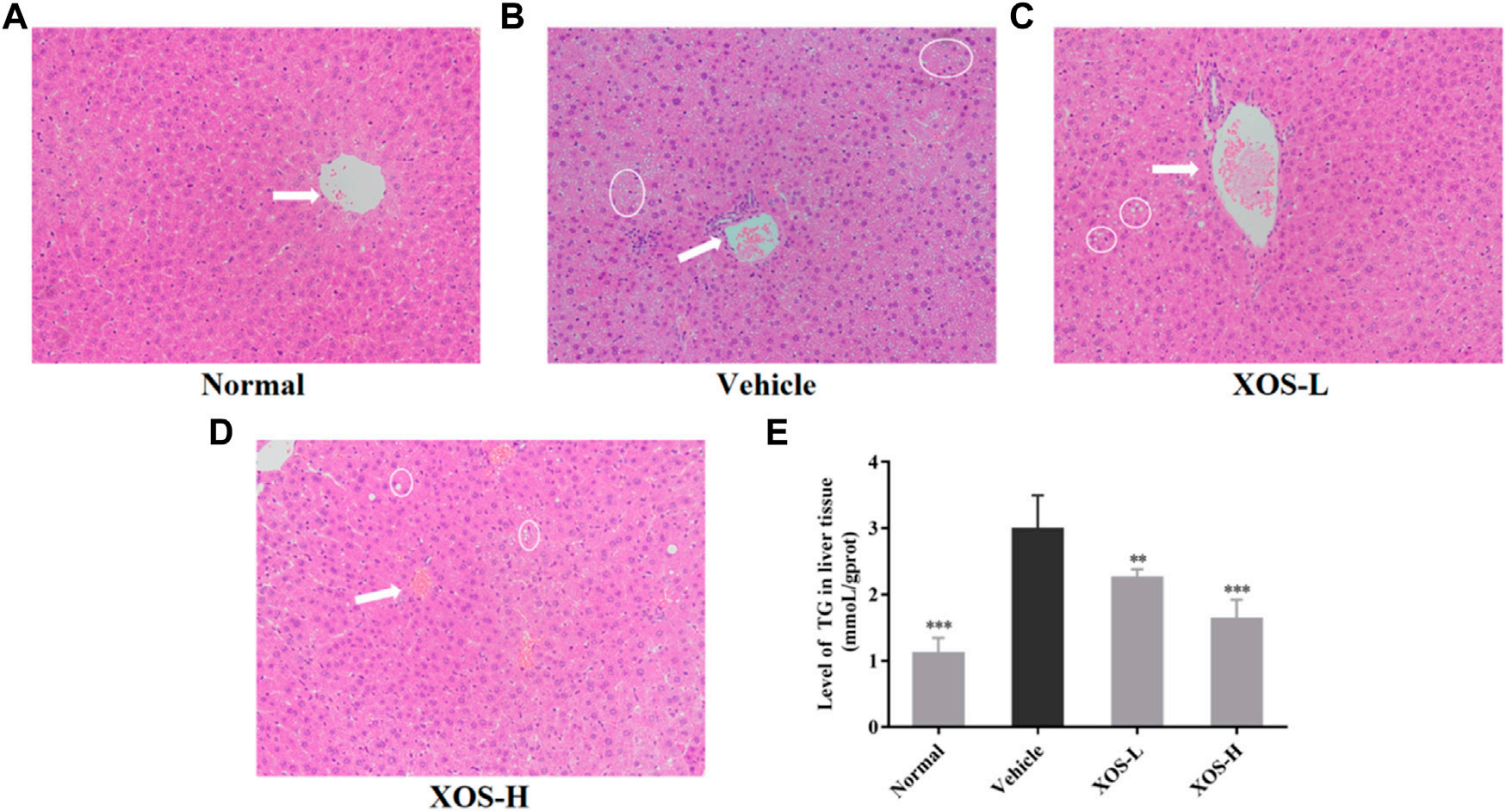
**(A–D)** Effect of XOS supplementation on H&E pathological observation of liver tissue in mice. Magnification 10×; **(E)** Level of TG in liver tissue. XOS-L: mice treat with XOS 250 mg/kg, XOS-H: mice treat with XOS 500 mg/kg. The arrow indicates the central vein, the circled indicates lipid drop. **p < 0.01, ***p < 0.001 compared with the Vehicle group.

No steatosis was detected in the normal group, and the tissue structure was clear (Figure 4A). At the same time, hepatocytes had abundant cytoplasm, with clear membranes, a regular arrangement, and large and normal nuclei at the center. Different shapes of lipid droplets could be seen in the liver cells of the vehicle group, and the boundaries between cells were blurred (Figure 4B). After XOS intervention, the density of fat vesicles in mouse liver cells reduced, and the lipid droplets were smaller, scattered, and sparse. The nucleus was located at the center of the cell, and its morphology and structure were basically normal, with a tendency to gradually return to those of normal liver cells (Figures 4C,D). Meanwhile, the determination of the TG content in the liver tissue also confirmed that a high-fat diet significantly increased (*p* < 0.05) the TG content in the liver. XOS intervention inhibited the accumulation of TG in the liver, and the effect of high-dose XOS was more obvious (Figure 4E). This showed that XOS improved liver lipid accumulation.

**FIGURE 5:**
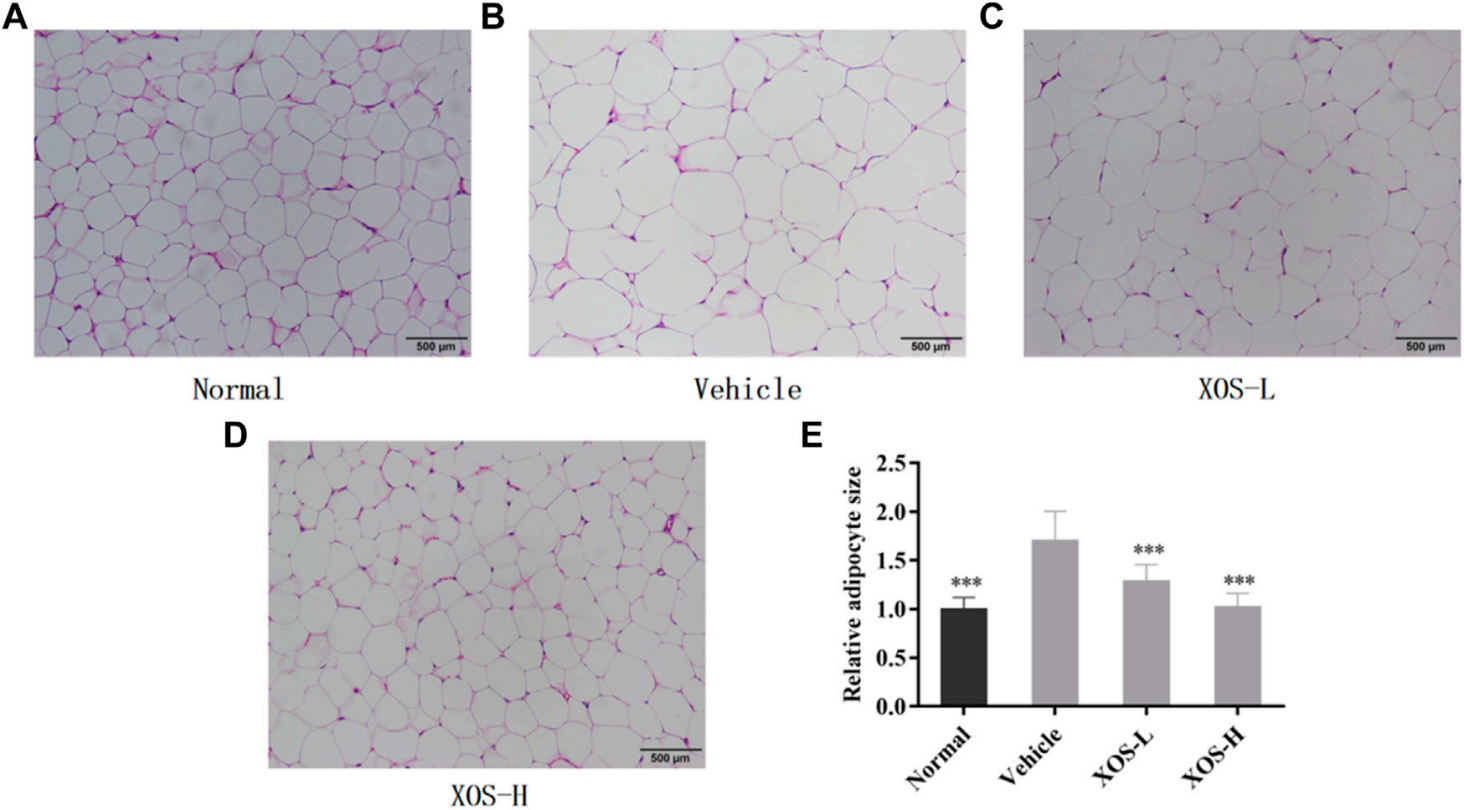
**(A–D)** Effect of XOS supplementation on H&E pathological observation of epididymal fat in mice; **(E)** Relative adipocyte size. Magnification 100×. Normal: XOS-L: mice treat with XOS 250 mg/kg, XOS-H: mice treat with XOS 500 mg/kg. ***p < 0.001 compared with the Vehicle group.

The fat cells in the epididymal fat in the normal group were uniform in size, neatly arranged, and dense. However, the fat cells in the vehicle group were hypertrophic and loosely arranged. Some cells were even twice as large as the fat cells in the normal group (Figures 5A–E). After intragastric administration of different doses of XOS to mice with hyperlipidemia, the appearance of epididymal fat cells was improved, the fat cells became smaller, and the structure was tighter. In particular, high-dose XOS significantly reduced adipocyte hypertrophy. Meanwhile, the results also confirmed that a high-fat diet significantly increased (*p* < 0.001) the adipocyte diameter, while XOS significantly improved this trend (*p* < 0.001), which was close to the results obtained in the normal group (Figure 5E).

### Expression of RNA in Mouse Epididymal Fat Tissue

**FIGURE 6:**
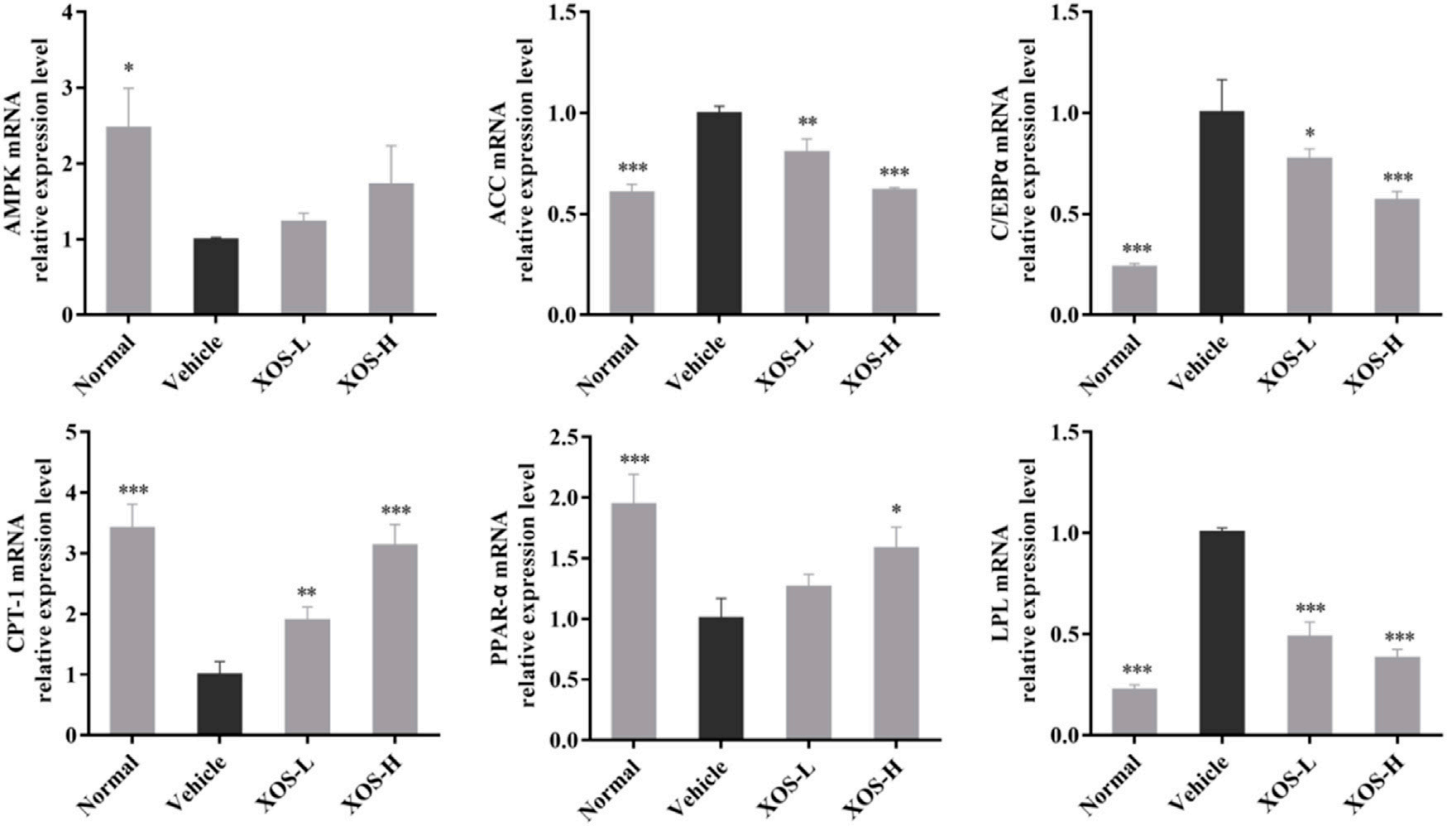
Effect of XOS supplementation on AMPK, ACC, C/EBPα, CPT-1, PPAR-α, and LPL mRNA expression in epididymal fat tissue of mice. XOS-L: mice treat with XOS 250 mg/kg, XOS-H: mice treat with XOS 500 mg/kg. *p < 0.05, **p < 0.01, ***p < 0.001 compared with the Vehicle group.

The mechanism of lipid-regulating activity of XOS was preliminarily discussed, and the gene expression of AMPK, ACC, CPT-1, C/EBPα, LPL, and PPAR-α in epididymal fat tissue was determined by RT-qPCR. In the normal mice samples, AMPK, CPT-1, and PPAR-α expression levels were the highest, while ACC, C/EBPα, and LPL expression levels were the lowest (Figure 6). The vehicle group mice samples completely showed the opposite trend. The intragastric XOS doses of 250 and 500 mg/kg reversed the gene expression in mice fed a high-fat diet and inhibited the downregulation of AMPK, CPT-1, and PPAR-α and the upregulation of ACC, C/EBPα, and LPL. Among these, high-dose XOS had a stronger effect, and the expression of related genes in mouse epididymal fat was closer to that in normal mice.

### Expression of RNA in Mouse Liver Tissue

**FIGURE 7:**
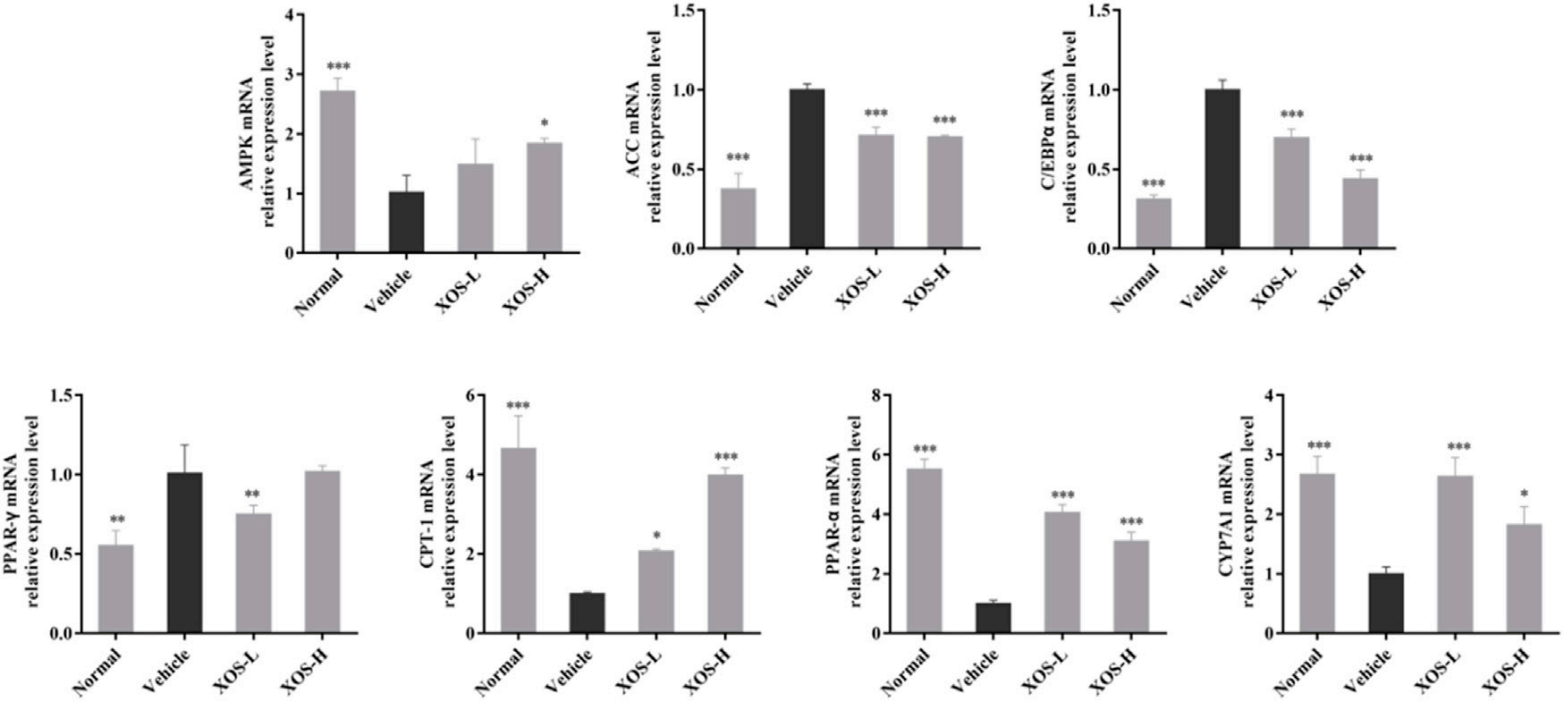
Effect of XOS supplementation on AMPK, ACC, C/EBPα, PPAR-γ, CPT-1, PPAR-α, and CYP7A1 mRNA expression in liver tissue of mice. XOS-L: mice treat with XOS 250 mg/kg, XOS-H: mice treat with XOS 500 mg/kg. *p < 0.05, **p < 0.01, ***p < 0.001 compared with the Vehicle group.

We studied the mRNA expression of genes involved in lipogenesis, fatty acid oxidation, and cholesterol metabolism in liver tissues (Figure 7). The AMPK mRNA expression was significantly reduced (*p* < 0.001) in the vehicle group compared with the normal group. After XOS gavage intervention, AMPK expression increased, indicating that XOS intervention activated the AMPK pathway and increased AMPK expression. Activating AMPK to regulate fatty acid metabolism might be related to the ability of AMPK to participate in the body’s decomposition and anabolism at the same time. The high-fat diet increased the expression of fatty acid synthesis–related genes, including ACC, C/EBPα, and PPAR-γ, but gavage XOS significantly reduced their expression. At the same time, the expression of fatty acid β-oxidation–related genes (PPAR-α and CPT-1) increased at the mRNA level after XOS intervention. In addition, a high-fat diet reduced the expression of the CYP7A1 gene for bile acid synthesis in mice. Supplementing XOS increased the expression of CYP7A1 and promoted the conversion of cholesterol into bile acids.

### Expression of RNA in Mouse Small Intestine Tissues

**FIGURE 8:**
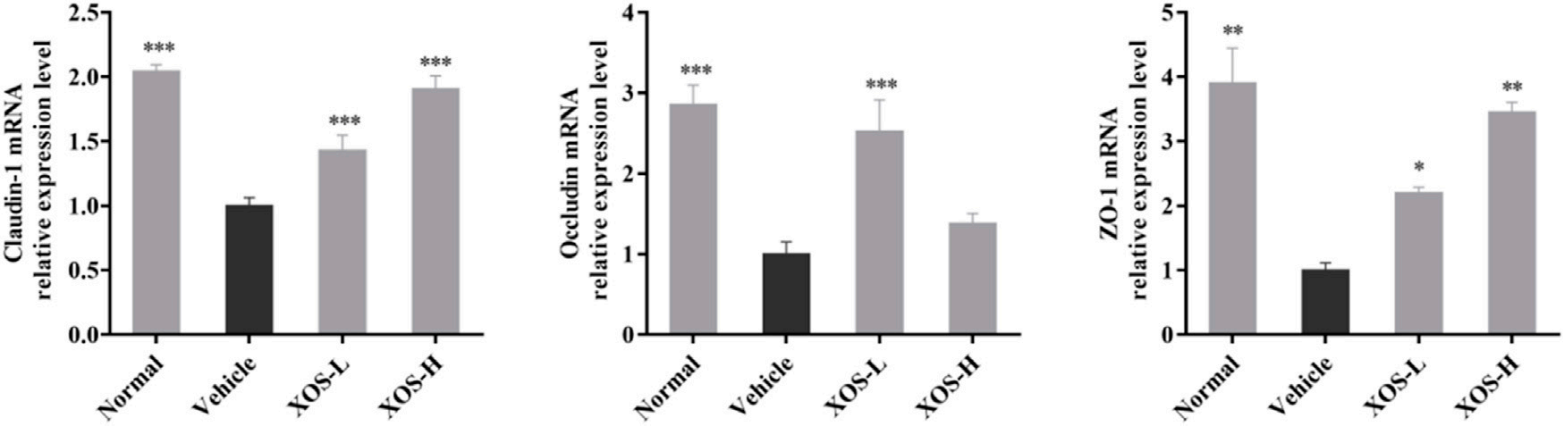
Effect of XOS supplementation on Claudin-1, Occludin and ZO-1 mRNA expression in small intestine tissue of mice. XOS-L: mice treat with XOS 250 mg/kg, XOS-H: mice treat with XOS 500 mg/kg. *p < 0.05, **p < 0.01, ***p < 0.001 compared with the Vehicle group.

The high-fat diet had a negative regulatory effect on the intestinal mucosa, increased the levels of cytokines that destroyed the barrier, and increased the number of species in the flora that destroyed the barrier. Therefore, the expression of intestinal TJ proteins occludin, claudin-1, and ZO-1 in experimental mice was detected by RT-qPCR. The results showed that the expression of occludin, claudin-1, and ZO-1 was lower in the intestine of the vehicle group compared with the other groups; different doses of XOS increased the expression of occludin, claudin-1, and ZO-1 (Figure 8).

### Protein Expression in Mouse Liver Tissues

**FIGURE 9:**
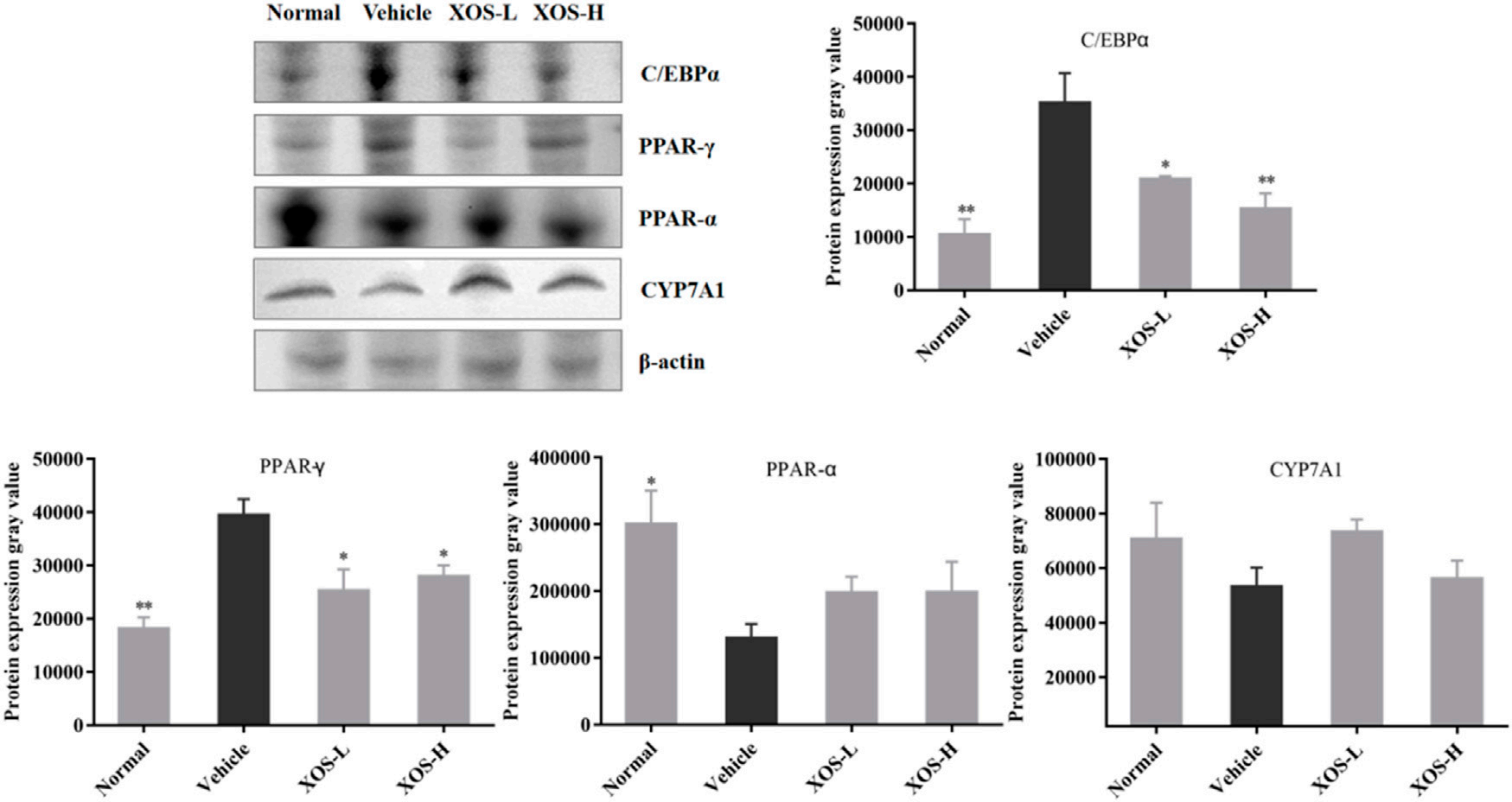
Effect of XOS supplementation on C/EBPα, PPAR-γ, PPAR-α, and CYP7A1 protein expression in liver tissue of mice. XOS-L: mice treat with XOS 250 mg/kg, XOS-H: mice treat with XOS 500 mg/kg. *p < 0.05, **p < 0.01 compared with the Vehicle group.

Western blot analysis detected the expression of proteins in the liver of mice (Figure 9). The high-fat diet decreased the protein expression of CYP7A1 and PPAR-α and increased the protein expression of PPAR-γ and C/EBPα. XOS-H intervention significantly inhibited the upregulation of PPAR-γ (*p* < 0.01) and C/EBPα (*p* < 0.001) expression, as well as the downregulation of PPAR-α expression. XOS had a tendency to increase the expression of CYP7A1, but the effect was not significant.

### Microbial RNA Expression in Mouse Feces

**FIGURE 10:**
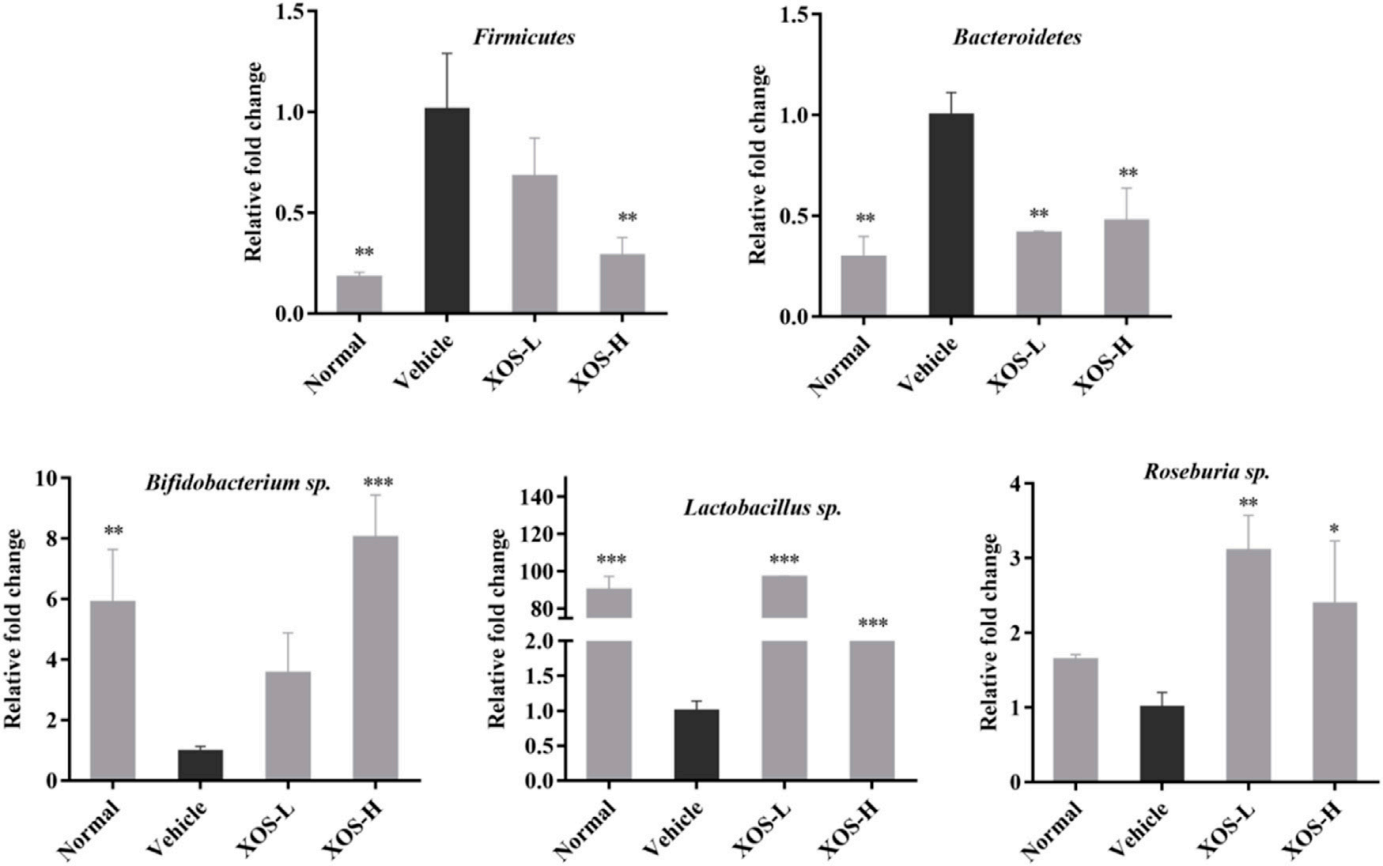
Effect of XOS supplementation on abundances of *Firmicutes*, *Bacteroidetes*, *Bifidobacterium* sp., *Lactobacillus* sp., *Roseburia* sp. XOS-L: mice treat with XOS 250 mg/kg, XOS-H: mice treat with XOS 500 mg/kg. *p < 0.05, **p < 0.01, ***p < 0.001 compared with the Vehicle group.

Using the total bacterial DNA as an internal reference, the relative expression results of *Firmicutes*, *Lactobacillus* sp., *Bifidobacterium* sp., *Roseburia* sp., and *Bacteroides* in the cecal content of each group of mice are shown in Figure 10. The RT-qPCR results showed that the high-fat diet increased the abundance of *Firmicutes* and, at the same time, decreased the abundance of *Bacteroides, Lactobacillus* sp., *Bifidobacterium* sp., and *Roseburia* sp. The abundance of *Firmicutes* increased significantly (*p* < 0.01) in the high-fat diet group, which was 5.55 times that in the normal diet group. The abundance of *Bacteroides, Lactobacillus* sp., *Bifidobacterium* sp., and *Roseburia* sp. decreased 0.43, 0.01, 0.17, and 0.61 times than that in the normal diet group, respectively. After XOS intervention, especially higher doses of XOS, the abundance of *Firmicutes* reduced 0.29 times, while the abundance of *Bacteroides*, *Lactobacillus* sp., and *Bifidobacterium* sp. increased 1.81, 63.33, and 8.06 times, respectively. However, the difference might not be statistically significant due to the insufficient number of samples.

## Discussion

Hyperlipidemia is accompanied by weight gain and abnormal lipid metabolism as an important pathological basis for a series of diseases such as atherosclerosis, hypertension, diabetes, and so forth (Teng et al., 2020). The activities of ALT and AST and the levels of LDL-C, HDL-C, TC, and TG in the serum of mice are often used as the clinical criteria for diagnosing hyperlipidemia (Chen et al., 2018). The abnormal increase in serum TC content leads to the disorder of arterial endothelial cell function, which accelerates the occurrence and development of cerebrovascular diseases, especially atherosclerosis (Nakamura et al., 2016). Increased serum TG content can inhibit the dissolution of fibrin in the blood, increase blood viscosity, promote thrombosis, and accelerate the development of atherosclerosis (Zhao et al., 2021). On the contrary, the main carrier of plasma sterols in the body is LDL-C. Cholesterol is often accumulated in vascular endothelial cells, which can easily cause atherosclerosis. At the same time, HDL-C has an important biological function, that is, it helps transport excess cholesterol from all parts of the body to the liver of specific organs (Wang et al., 2010; Zhang et al., 2017). After intragastric intervention with XOS, the mice lost weight, the weight of epididymal fat tissue was reduced, and the fat cells became smaller and were arranged more regularly. Moreover, the serum TG, TC, and LDL-C levels significantly decreased, and the activities of ALT and AST in the serum of mice with hyperlipidemia improved, indicating that XOS could effectively accelerate the decomposition of cholesterol, reduce the levels of lipid peroxidation, and achieve the purpose of regulating and reducing blood lipid levels. However, the study (Long et al., 2019) found that giving mice dietary XOS supplements had no significant changes in blood and liver lipids including cholesterol, and the composition of cecum microbiota in mice has changed significantly.

A high-fat diet changes intestinal flora and destroys the intestinal barrier. TJ proteins and mucus secreted by intestinal epithelial cells play the role of the first line of defense in preventing intestinal flora translocation. They are also the main guarantee for the integrity of the intestinal mucosal barrier (Bu et al., 2020). TJ proteins can be divided into transmembrane proteins and cytoplasmic proteins according to their existing sites. The discovered transmembrane proteins include occludin and claudin, while the cytoplasmic proteins mainly include ZO-1, rab3b, and so forth (Krug et al., 2014). Intestinal mucosal barrier dysfunction is one of the common features of hyperlipidemia, mainly including increased mucosal permeability, damage to intestinal epithelial TJ protein, sparse shedding of intestinal villi, increased pathogenic bacteria, and imbalance of intestinal flora, suggesting that the damage to the intestinal mucosal barrier might be an important mechanism of hyperlipidemia (Wigg et al., 2001). The study on Sprague–Dawley rats with hyperlipidemia also confirmed that the expression of ZO-1 and occludin protein in the intestine decreased and the permeability of intestinal mucosa increased (Lee et al., 2015). In this study, we found that the expression of ZO-1, occludin, and claudin-1 genes decreased significantly in the high-fat diet group. XOS intervention improved the expression of TJ protein genes and improved the damage to the intestinal mucosal barrier. This study confirmed that a high-fat diet caused changes in the intestinal flora of mice and damaged the intestinal barrier, showing that the results decreased the expression of ZO-1, occludin, and claudin-1, and more *Firmicutes* and fewer *Bacteroides*. XOS supplementation improved the flora structure and enhanced the effect of *Bacteroides*. *Bacteroides* and *Firmicutes* contain most of the genes related to lipid accumulation (Long et al., 2019). Similarly, a study found that compared with the normal control group, the number of *Firmicutes* and *Enterobacteriaceae* in the intestinal tract of mice in the high-fat-diet group increased, while *Lactobacillus* sp. and *Bifidobacterium* sp. decreased (Sarma et al., 2018). These results showed that a high-fat diet may affect the growth of probiotics in the intestines, and the reduction in probiotics reduces the conversion and utilization of cholesterol, increases blood lipids, and causes chronic inflammation throughout the body. The results of Finegold et al.’s study are also similar to the results of this study (Finegold et al., 2014). The study measured the stool of healthy people who supplemented with XOS and found that the intake of XOS has an effect on the beneficial gastrointestinal microbiota, especially increasing the content of *Bififidobacterium*, but has no significant effect on *Lactobacillus.*

Changes in the composition of the flora can affect various metabolic organs, such as the liver and adipose tissue metabolism. When the synthesis, transport, and decomposition of fatty acids and triglycerides in tissues are hindered, lipids may be deposited in the liver and gradually form fatty livers or accumulate in subcutaneous tissues and may also cause inflammation in the body (Tietge, 2014; Chen et al., 2018). A recent study found that TNF-α directly or indirectly interfered with and hindered the metabolism of triglycerides and cholesterol, thereby further affecting the body’s lipid metabolism and finally regulating blood lipid levels to a certain degree (Jung and Choi, 2014). IL-6 helps synthesize LDL receptors on the surface of macrophages. It also promotes the uptake of LDL by macrophages, thereby increasing the level of LDL-C and lipid deposition, and indirectly accelerates the formation of atheroma (Wang et al., 2014). On the contrary, IL-10, as an anti-inflammatory cytokine, inhibits the expression of inflammatory factors, such as TNF-α, IL-6, and IL-1β, through activated macrophages and also has a certain regulatory effect on blood lipids (Han et al., 2010). Vitseva et al. also believed that a correlation existed between inflammatory factors and the mechanism of blood lipid–related metabolic dysfunction (Vitseva et al., 2008). After intragastric treatment with XOS, the inflammation of the body improved, showing an increased level of IL-10 and reduced levels of TNF-α, IFN-γ, and IL-6.

The liver is the central organ of the body’s lipid metabolism, and the accumulation of lipids in the liver played an important role in the study of hyperlipidemia (Chen et al., 2018). We measured the expression of lipid synthesis–and lipolysis-related genes to explore further the mechanism of XOS regulating serum lipids in mice with hyperlipidemia mice and improving lipid accumulation in the body. The epididymal fat is an important place for triglyceride accumulation and an important organ that secretes many adipokines (Corton et al., 1994). XOS may improve the lipid metabolism disorder in mice through the following ways: 1) AMPK pathway is a classic pathway that regulates lipid metabolism. Once the AMPK pathway is activated, the expression of AMPK-α increases. The activated AMPK can directly phosphorylate the Ser-79 site of ACC1. The Ser-218 site of ACC2 inhibits the activity of ACC, thereby reducing the content of malonyl-CoA, fat synthesis, and fat accumulation (Corton et al., 1994; Schober et al., 2013). A previous study showed that phenolic glycoside reduced lipid accumulation by promoting the phosphorylation of ACC and AMPK in liver fat cells (Bao et al., 2020). This was consistent with the results of the present study. The high-fat diet reduced the AMPK mRNA expression, and the relative expression increased after XOS intervention, indicating that the intervention activated the AMPK pathway and increased the expression of AMPK, inhibiting the expression of ACC and fat synthesis. 2) C/EBPα-mediated lipid synthesis: PPAR-γ and C/EBPα are transcription factors that regulate the differentiation of adipocytes to produce triglyceride lipid droplets. As adipocytes differentiate, epididymal fat continues to accumulate, and the contents of PPAR-γ and C/EBPα increase. The study concluded that artesunate inhibited adipogenesis in 3T3-L1 preadipocytes by downregulating the relative expression of C/EBPa and PPAR-γ (Jang, 2016). In this experiment, intervention in the XOS group reduced the mRNA and protein expression of C/EBPa and PPAR-γ in liver tissues, but not in a dose-dependent manner. 3) Upregulation of PPAR-α-mediated fatty acid oxidation metabolism: PPAR-α played an important role in lipid oxidation metabolism and could activate lipid oxidation gene CPT-1, promote lipid decomposition, and reduce lipids in the body accumulation. CPT-1 is mainly responsible for transporting fatty acids in the liver to the mitochondria. Qin Hong found that capsaicin could upregulate the relative expression of the CPT-1 gene in the epididymal fat of mice, thereby promoting the decomposition of triglycerides and reducing the accumulation of triglycerides (Hong et al., 2015). Consistent with these findings, the gene expression of PPAR-α was upregulated in XOS groups, thereby activating the expression of CPT-1, accelerating the β-oxidation of fatty acids in the liver, and reducing the deposition of body lipids. 4) CYP7A1-mediated cholesterol metabolism: CYP7A1 is the rate-limiting enzyme that converts cholesterol into bile acid in the liver (Pandak et al., 2001). We measured the gene and protein expression of CYP7A1 in the liver and found that it was suppressed by a high-fat diet. However, supplementation with XOS upregulated the expression of CYP7A1, accelerating the metabolism of cholesterol.

## Conclusion

The results showed that XOS improved hyperlipidemia. The protective effect of XOS was mainly through 1) increasing the abundance of intestinal flora and simultaneously promoting the growth of beneficial intestinal flora. 2) The intestinal–liver axis–dependent mechanism increased the strength of the intestinal barrier, regulated liver lipid metabolism disorders, and reduced inflammation in the body. These results indicated that XOS had the therapeutic potential to improve hyperlipidemia Câ ndido et al., 2017.

## Data Availability

The original contributions presented in the study are included in the article/Supplementary Material, further inquiries can be directed to the corresponding author.
